# Crystal Structures of Carbamate Kinase from *Giardia lamblia* Bound with Citric Acid and AMP-PNP

**DOI:** 10.1371/journal.pone.0064004

**Published:** 2013-05-20

**Authors:** Kap Lim, Liudmila Kulakova, Andrey Galkin, Osnat Herzberg

**Affiliations:** 1 Institute for Bioscience and Biotechnology Research, University of Maryland, Rockville, Maryland, United States of America; 2 Department of Chemistry and Biochemistry, University of Maryland, College Park, Maryland, United States of America; Russian Academy of Sciences, Institute for Biological Instrumentation, Russian Federation

## Abstract

The parasite *Giardia lamblia* utilizes the L-arginine dihydrolase pathway to generate ATP from L-arginine. Carbamate kinase (CK) catalyzes the last step in this pathway, converting ADP and carbamoyl phosphate to ATP and ammonium carbamate. Because the L-arginine pathway is essential for *G. lamblia* survival and absent in high eukaryotes including humans, the enzyme is a potential target for drug development. We have determined two crystal structures of *G. lamblia* CK (*gl*CK) with bound ligands. One structure, in complex with a nonhydrolyzable ATP analog, adenosine 5′-adenylyl-β,γ-imidodiphosphate (AMP-PNP), was determined at 2.6 Å resolution. The second structure, in complex with citric acid bound in the postulated carbamoyl phosphate binding site, was determined in two slightly different states at 2.1 and 2.4 Å resolution. These structures reveal conformational flexibility of an auxiliary domain (amino acid residues 123–170), which exhibits open or closed conformations or structural disorder, depending on the bound ligand. The structures also reveal a smaller conformational change in a region associated the AMP-PNP adenine binding site. The protein residues involved in binding, together with a model of the transition state, suggest that catalysis follows an in-line, predominantly dissociative, phosphotransfer reaction mechanism, and that closure of the flexible auxiliary domain is required to protect the transition state from bulk solvent.

## Introduction

**Figure 1:**
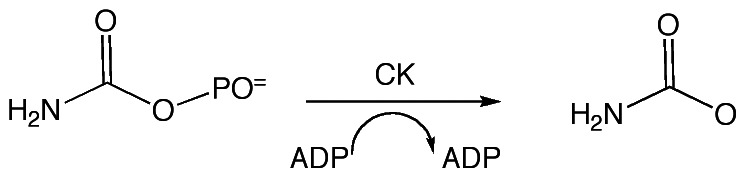
The reaction catalyzed by carbamate kinase.

Carbamate kinase (CK; EC 2.7.2.2, ATP:carbamate phosphotransferase) reversibly converts ADP and carbamoyl phosphate into ATP and ammonium carbamate (Fig. 1). The enzyme functions in the L-arginine dihydrolase pathway, operative primarily in some bacteria but also in the enteric protozoan parasite, *Giardia lamblia*. In contrast, CK is absent in high eukaryotes including Human. Since *Giardia* lacks the enzymes of the citric acid cycle and the oxidative phosphorylation pathway, the L-arginine dihydrolase and glycolytic pathways supply *G. lamblia* with ATP [1]. In addition, the L-arginine hydrolase pathway deprives host intestinal epithelial cells of arginine for nitric oxide biosynthesis, thereby dampening the innate immunity defenses [2], [3]. Northern blotting revealed that the *G. lamblia* CK (*gl*CK) gene was primarily expressed in *Giardia* trophozoites and that the mRNA level significantly decreased after encystation [4]. The *gl*CK gene is also one of eight genes whose expressions are upregulated in neomycin selected *Giardia* cell lines [5], suggesting its importance under stress conditions. To validate *gl*CK as a potential drug target, we used RNAi to knock down the *gl*CK gene, which showed that the enzyme is essential for the survival of the organism under optimal laboratory growth conditions (*Giardia* possesses two nuclei and gene knockout is impractical) [6]. These data establish *gl*CK as a potential target for drug development.

Previously, we have determined the crystal structure of *gl*CK at 3.0 Å resolution [6], which revealed a large active site crevice within a core α/β domain. A region comprising 30 amino acid residues flanking the active site was largely disordered in that crystal structure suggesting a possible role for enzyme conformational transition during catalysis. Here, we report two enzyme-ligand crystal structures determined at higher resolutions than the earlier structure. We identify residues that are likely to play key roles during catalysis, and analyze a range of ligand-dependent protein conformational flexibility that protects the catalytic site from bulk solvent during the chemical reaction. One structure contains a bound non-hydrolyzable ATP analog, 5′-adenylyl-β,γ-imidodiphosphate (AMP-PNP), and the second structure (determined in two slightly different crystal forms) contains citric acid bound in the postulated carbamoyl phosphate site. These structures enable comparison with structures of ligand-bound CK from *Enterococcus faecalis* (*ef*CK) [7] and the modeling of the transition state complex.

## Materials and Methods

### Protein Preparation and Crystallization

The *gl*CK gene was cloned into the pDEST-HisMBP expression vector [8], and produced in *E. coli* strain BL21(DE3)Star as a TEV protease-cleavable, His-tagged, maltose binding protein (MBP) fusion product [6]. The cleaved and purified protein was concentrated to 30 mg/mL in solution containing 50 mM Tris-HCl (pH 8.0), 0.1 M NaCl, 5 mM MgCl_2_, and 1 mM DTT. DTT was required to prevent the protein from aggregation. The protein sample was stored in aliquots at −80°.

The *gl*CK was crystallized using the vapor-diffusion method in hanging drops. The protein solution was mixed with an equal volume of mother liquor containing 0.4 M ammonium citrate dibasic (pH 5.0), and 21% (w/v) polyethylene glycol (PEG) 3350. These *gl*CK crystals contained citric acid bound in the active site (designated hereafter as *gl*CK*-*citrateL). Crystals appeared within 1 to 3 days. For X-ray diffraction data acquisition, the crystals were transferred to mother liquor supplemented with 20% (v/v) glycerol and flush-cooled in liquid nitrogen.

A second *gl*CK structure, also with bound citrate, was obtained after an unsuccessful attempt to soak a *gl*CK*-*citrateL crystal with the drug Disulfiram, recently identified as a *gl*CK inhibitor [9]. Flush cooling of the crystal led to shrinkage of the *b* unit cell dimension by >6%, and improvement in the diffraction resolution from 2.4 to 2.1 Å (Table 1). This structure is designated hereafter as *gl*CK-citrateS. Finally, soaking a *gl*CK*-*citrateL crystal with 50 mM AMP-PNP for 4 hours displaced the citric acid and yielded a structure with AMP-PNP bound in the active site (designated hereafter *gl*CK-AMPPNP).

**Table 1:** X-ray data collection and structure refinement statistics.

Crystal	*gl*CK-citrateL	*gl*CK-citrateS	*gl*CK-AMPPNP
*Data collection*			
Cell dimension (Å, °)	*a* = 69.9, *b* = 92.7, *c* = 101.8, β = 106.2	*a* = 69.9, *b* = 86.4, *c* = 102.0, β = 106.5	*a* = 70.6, *b* = 97.1, *c* = 102.1, β = 106.7
Wavelength (Å)	1.5418	1.5418	1.0332
Resolution (Å)	2.4	2.1	2.6
No. of observed reflections	162,457	202,519	130,375
Completeness (%)^a^	99.4 (99.9)	99.6 (97.5)	99.2 (99.2)
No. of unique reflections	48,667	67,363	40,467
*R*_merge_ b	0.097 (0.355)	0.092 (0.322)	0.093 (0.272)
<I/σ (I)>	7.2 (3.3)	7.9 (2.9)	8.1 (3.6)
Redundancy	3.3 (3.4)	3.0 (2.8)	3.2 (3.1)
*Refinement*			
No. of reflections used	48,666	67,363	40,450
No. of protein atoms	9,448	9,313	8,960
No. of ligand atoms	52 (citrate)	52 (citrate)	124 (AMPPNP)
No. of water atoms	275	700	475
*R*_cryst_ c	0.218 (0.302)	0.220 (0.285)	0.216 (0.275)
*R*_free_ d	0.278 (0.367)	0.288 (0.380)	0.298 (0.344)
RMSd from ideal geometry			
Bond length (Å)	0.012	0.013	0.010
Bond angle (°)	1.4	1.4	1.3
ΔB bonded (Å^2^)	1.2	1.4	1.7
Wilson B (Å^2^)	31	24	18
Average B factor (Å^2^			
Average B factor (Å^2^)			
Protein	44	31	24
Ligand	47	35	28
Water	35	33	21
Ramachandran plot (%)^e^	86.0, 13.7, 0.3, 0.0	88.5, 10.9, 0.6, 0.0	85.9, 13.9, 0.2, 0.0

a The values in parentheses are for the highest resolution shell, 2.40–2.49 Å for *gl*CK-citrateL, 2.10–2.15 Å for *gl*CK-citrateS, and 2.60–2.73 Å for *gl*CK-AMPPNP.

b *R_merge_ = *∑*_hkl_* [(∑*_j_* | *I_j_* –<*I*>| )/∑*_j_* | *I_j_* | ].

c *R*_cryst_ = ∑*_hkl_* | |*F_o_*| – |*F_c_*| |/∑*_hkl_* |*F_o_*|, where *F_o_* and *F_c_* are the observed and calculated structure factors, respectively.

d *R*_free_ is computed using 2,009 randomly selected reflections omitted from the refinement for *gl*CK-citrateL, 2,670 for *gl*CK-citrateS, and 1,616 for *gl*CK-AMPPNP.

e Ramachandran plot categories are most favored, allowed, generously allowed, and disallowed.

### Data Collection and Refinement

X-ray diffraction data for both *gl*CK-citrate crystals were acquired using an R-AXIS IV++ image-plate detector, mounted on a Rigaku rotating-anode MicroMax-007 X-ray generator (Rigaku MSC Inc). The diffraction data for the AMP-PNP soaked *gl*CK crystal were collected at the GM/CA-CAT ID beam line at the Advanced Photon Source, Argonne National Laboratory, Illinois. The beam line was equipped with a MARmosaic 300 CCD detector (Marresearch GmbH).

Diffraction data were processed with the XDS program [10]. The previously determined *gl*CK crystal structure was used as the model for the calculation of the initial difference Fourier electron density map [6]. Rigid body minimizations and refinements of the structures were carried out with the Phenix program [11]. Anisotropic scaling, bulk solvent correction, and isotropic temperature factors were used. TLS refinement did not improve the *R* values and therefore was not used. Water molecules were assigned using peaks in the *F*_o_ – *F*_c_ difference Fourier map with electron density >3σ as the acceptance criteria. Model building and structure modifications were performed with the Coot interactive graphics program [12].

Figures were generated with Raster3D linked to Molscript [13], [14]. The coordinates and structure factors were deposited in the Protein Data Bank (entry codes 4JZ9 for *gl*CK-citrateL, 4JZ8 for *gl*CK-citrateS, and 4JZ7 for *gl*CK-AMPPNP.

## Results and Discussion

### Conformational Flexibility of CK

**Figure 2:**
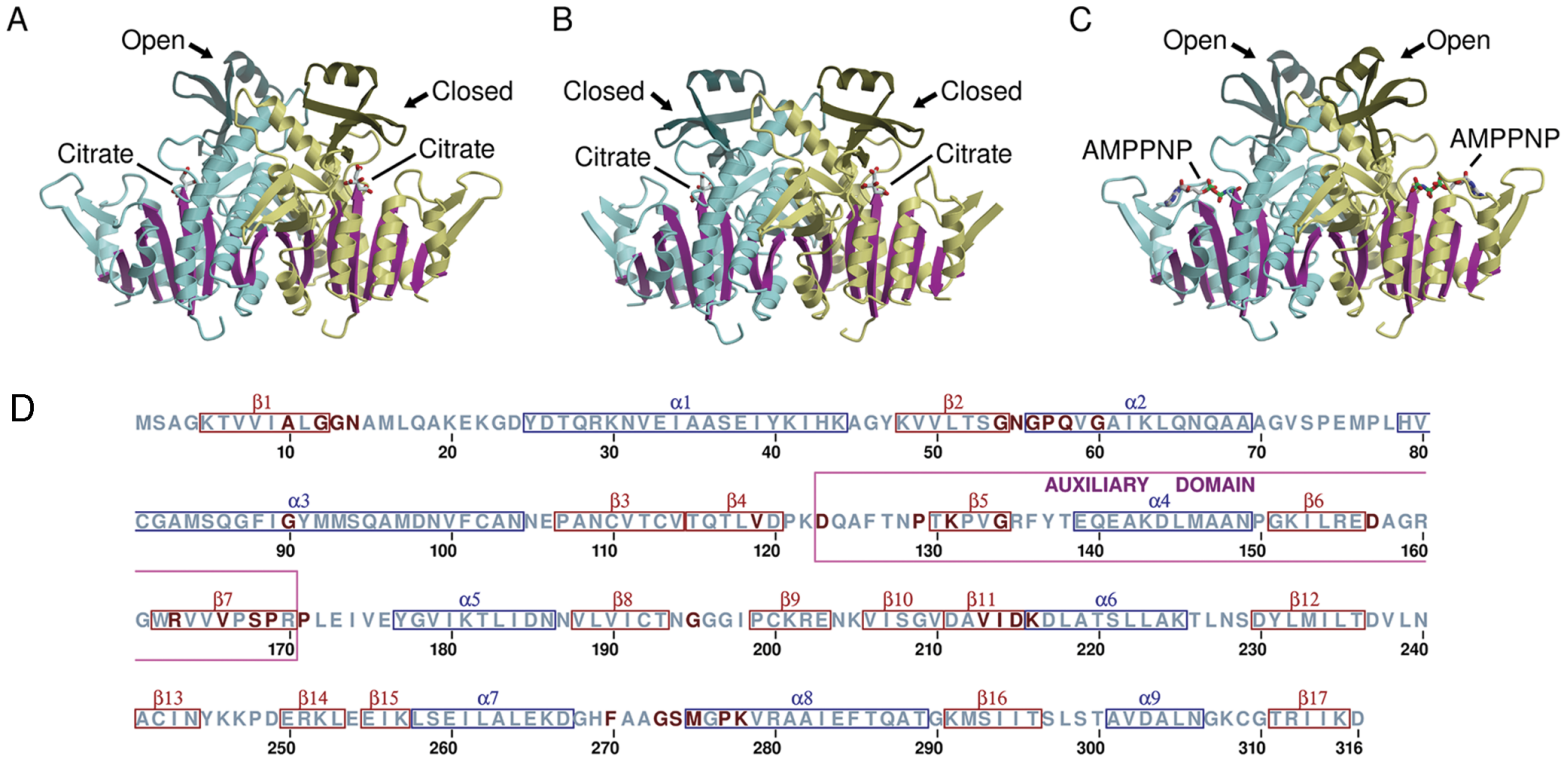
Ribbon depiction of dimeric ***gl*** **CK structures.** The flexible auxiliary domains and the core α/β domains are highlighted in different colors. Bound ligands are shown as stick models. (A) One homodimer of the *gl*CK-citrateL structure contains one auxiliary domain in an open conformation and the second in a closed conformation. (B) The second homodimer of the *gl*CK-citrateL structure and both homodimers of the *gl*CK-citrateS structure exhibit the auxiliary domains only in the closed conformation. (C) The homodimer in the *gl*CK-AMPPNP crystal asymmetric unit that exhibits the open conformation. The auxiliary domains of the second homodimer are disordered, which is not shown. (D) Amino acid sequence conservation of *gl*CK based on multiple alignment of the top 100 sequences identified using the BLAST protein sequence homology search [28]. Multiple sequence alignment was performed with ClustalW [29]. Invariant residues are colored in red. The N-terminus of two CKs in the non-redundant sequence database are truncated, thus only 98 sequences were used to define the first three invariant residues. Secondary structure units are boxed. β-strands and α-helices are show in red and blue colors, respectively, and in addition, the auxiliary domain is boxed in magenta color.

CK assembles into homodimers, consistent with the oligomeric form detected in solution (Fig. 2). Two homodimers occupy the crystal asymmetric unit. Each subunit contains an eight-stranded β-sheet surrounded by three α-helices on one side and four α-helices on the other side of the β-sheet. This is a modified “Rossmann fold” organization with two additional β-strands at one edge of the β-sheet running antiparallel to the remaining six parallel β-strands [6]. The dimer interface is formed along the edge of the core Rossmann fold and also involves an additional α-helix that protrudes from this core. The new structures reveal an auxiliary domain, comprising a short β-strand followed by an α-helix and a β-hairpin (amino acids 123–170; Fig. 2B), which was mostly disordered in the previously reported *gl*CK structure [6]. Depending on the bound ligand, the new crystal structures reveal this auxiliary domain in two different orientations with respect to the core domain. The large conformational changes, induced by soaking with ligands, are tolerated within the *gl*CK crystals because the auxiliary domains are located close to crystal solvent channels. These conformational changes were accompanied by substantial changes to the crystal’s *b* cell dimension (Table 1). The *b* unit cell expanded by 5% upon soaking with AMP-PNP, and shrank by 8% upon the non-productive soaking with Disulfiram (Table 1). Thus, this pliable crystal form enables the sampling of multiple conformations while maintaining a reasonably good diffraction quality. The structural flexibility of the auxiliary domain is reflected in the higher crystallographic temperature factors (Bs) when compared to the entire structure. The average overall B values for *gl*CK-citrateL and *gl*CK-citrateS are 44 Å^2^ and 31 Å^2^, respectively (calculated for the four molecules in the asymmetric unit). By contrast, the average B values for the corresponding auxiliary domains are higher, at 63 Å^2^ and 42 Å^2^. For the *gl*CK-AMPPNP structure, the overall B value is 24 Å^2^, lower than that of two well-defined auxiliary domains at 63 Å^2^. The remaining two auxiliary domains within the asymmetric unit are associated with weak electron density that cannot be interpreted.

**Figure 3:**
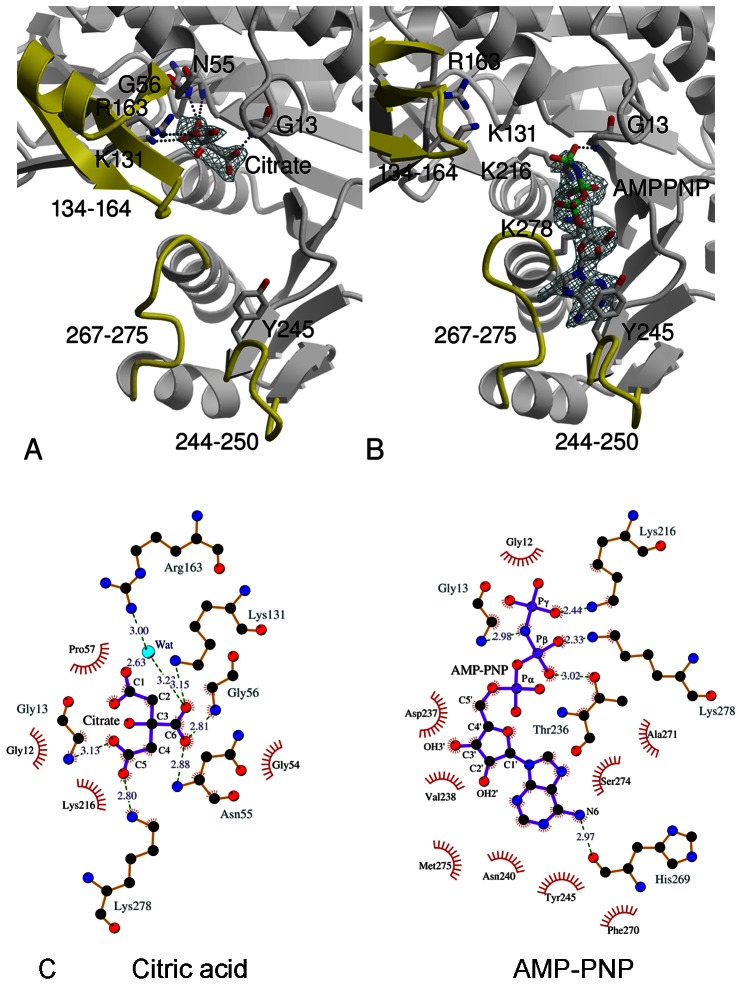
Ligands bound to the *gl*CK active site and their key interactions. (A) Citric acid. (B) AMP-PNP. The auxiliary domain (colored yellow) is in the closed conformation in (A) and in the open conformation in (B). Note the conformational differences of the loops 244–250 and 267–275 (colored yellow) with and without the bound AMP-PNP, leading to the stacking of Tyr245 above the adenine ring of AMP-PNP. The difference Fourier maps with the coefficients *F*_o_−*F*_c_, omitting the ligand from the model, are contoured at 2.5σ level. (C) Schemes and atom numbering of the ligands and their interactions with the protein. The figure was generated using the program LIGPLOT [30].

A citrate ion occupies each subunit of the *gl*CK homodimer in both *gl*CK-citrateS and *gl*CK-citrateL complexes (Figs. 2A & 3A). The main difference between the two structures is in the conformational state of one of the auxiliary domains. In the *gl*CK-citrateS structure, all four auxiliary domains in the asymmetric unit are placed in proximity to the bound citrate, a state referred to as the closed conformation. In contrast, only three auxiliary domains in the crystal asymmetric unit of the *gl*CK-citrateL structure adopt the closed conformation and one domain is placed more remotely from the citrate ligand, a state referred to as the open conformation.

In the *gl*CK-AMPPNP structure, the citrate ions were displaced by the AMP-PNP because of partial overlap at the location of the γ-phosphate moiety (Fig. 3A&B). Of the two homodimers in the asymmetric unit, the auxiliary domains of only one homodimer adopt the open conformation while those in the second homodimer are structurally disordered.

The different conformational states of the auxiliary domain are accommodated within the same crystal form by changes in the spatial relationship between the two dimers in the asymmetric unit. Compared with the *gl*CK-citrateL structure, one dimer in the *gl*CK-AMPPNP crystal asymmetric unit is rotated by 3.0° and translated by 1.6 Å relative to the partner dimer. The corresponding values between *gl*CK-citrateL and *gl*CK-citrateS are smaller, 0.3° and 0.6 Å, consistent with a changed disposition of only a single auxiliary domain in the crystal asymmetric unit.

Flexibility of the auxiliary domain has been observed in two previous crystal structures of *Enterococcus faecalis* CK (*ef*CK), one with bound ADP and Mg2^+^ and the second with bound SO_4_^2–^ [7]. Each of these two structures was determined in a different crystal form. In particular, the *ef*CK-SO_4_^2–^ structure exhibited the closed conformation and was associated with extensive crystal contacts. In contrast, the three *gl*CK complexes are accommodated within the same crystal framework. Because crystal contacts do not drive the conformational changes, the *gl*CK structures substantiate the proposed role of conformational flexibility during substrate binding and reaction pathway.

In addition to the *gl*CK and *ef*CK structures, three more CK or CK-like crystal structures have been deposited in the PDB. None of these exhibit the closed conformation. The structure of the enzyme from *Pyrococcus furiosus* contains ADP and exhibits the auxiliary domain in an open conformation [15]. The *Mycoplasma penetran* CK structure contains two sulfate ions in the active site, one in the carbamoyl phosphate binding site and the other in the nucleotide’s β-phosphate site. The auxiliary domain in this crystal structure is disordered [16]. Finally, a structure of CK from *Aeropyrum pernix K1* has been determined by the Riken Structural Genomics team but has not been published (PDB entry code 2E9Y). This structure does not contain any bound ligand and the auxiliary domain exhibits the open conformation.

The relative disposition of the auxiliary domain in the open and closed conformation of *gl*CK was analyzed using the program DynDom [17]. The conformational transition between the open and closed conformation within the *gl*CK-citrateL protomers was modeled by a rigid body rotation of 44° (with a small translational component of 1.2 Å) around an effective hinge axis that runs perpendicular to a plane at the base of the domain (defined by residues 126–131 and 166–168). Similarly, the transition from the closed conformation in the *gl*CK-citrate structures to the open conformation in the *gl*CK-AMPPNP structure can be described by 36° and 31° rotations and 1.3 Å and 0.2 Å translations around effective hinge axes for the two structurally defined auxiliary domains.

A similar conformational transition was reported for the auxiliary domain in *ef*CK with a 33° rotation about the effective hinge axis [7]. Comparison of the *gl*CK and *ef*CK auxiliary domains was facilitated by alignment of the secondary structure units of the core domains (161 of the 316 residues align with root-mean-squares deviation (RMSD) of 0.6–0.7 Å between α-carbon atoms). For the *gl*CK-AMPPNP and *ef*CK-ADP structures, the orientations of the respective auxiliary domains differ on average by a 16° rotation. For the closed conformations, the orientations of the respective auxiliary domains differ on average by an 11° rotation in the *gl*CK-citrateS and *ef*CK-SO_4_^2+^ structures. The differences in the open conformations may simply reflect the multiple orientations that the auxiliary domain can adopt in the absence of substrate or substrate analog. However, the difference in the orientation of the auxiliary domain in the closed conformational state is probably due to the different substrate analogs (citrate vs. SO_4_^2+^), each of which imposes distinct spatial constraints compared with the true substrate, carbamoyl phosphate.

### Nucleotide Binding Site

AMP-PNP binds in a pocket flanked by amino acid residues 11–13, 235–250, and 267–278, (Fig. 3B). It exhibits the same interactions whether CK’s auxiliary domain adopts the open conformation or is disordered, as the nucleotide does not interact with the auxiliary domain. An AMP-PNP molecule binds to each of the four subunits within the crystal asymmetric unit. Consistent with the amino acid sequence homology analysis, the key protein-nucleotide interactions in the *gl*CK-AMPPNP structure are conserved with those seen in the *ef*CK-ADP structure [7] with a few notable exceptions. The following interactions are common to both structures. The loop comprising amino acid residues 245–250 undergoes a conformational transition upon AMP-PNP binding such that the side chain of Tyr245 (Tyr238 in *ef*CK) stacks against the adenine ring and its hydroxyl group forms a hydrogen bond with the ribose O2′ hydroxyl group (see Fig. 3C for atom numbering and protein-ligand interactions). Although this tyrosine residue is sometimes replaced in other carbamate kinases by a phenylalanine or tryptophan, these replacements still permit aromatic-aromatic interactions.

Met275 (Met268 in *ef*CK) side chain stacks against the opposite face of the adenine moiety. Interestingly, other nucleotide binding proteins also exhibit methionine side chains that are placed to interact with ATP in a similar manner, for example carbamoyl phosphate synthetase [18] and acetylglutamate kinase [19].

Additionally, Val238 (Val231 in *ef*CK) forms a van der Waals contact with the ribose C3′. Thr236 Oγ atom (Thr229 in *ef*CK) forms a hydrogen bond with one of the oxygen atoms of the β-phosphate. The loop encompassing residues 267–275 adjusts such that the backbone carbonyl oxygen of His269 (His262 in *ef*CK) forms a hydrogen bond with N6 of the adenine ring. Ser274 (Ser267 in *ef*CK) shifts away from the ribose site to form a van der Waals interaction with the ribose O4′. Lys278 (Lys271 in *ef*CK) forms a salt bridge with the β-phosphate. Finally, the nitrogen atom of the AMP-PNP γ-imidophosphate interacts with the backbone amide nitrogen atom of Gly13 (Gly11 in *ef*CK).

Present in *gl*CK-AMPPNP but absent in the *ef*CK-ADP structure is a hydrogen bond between the O3′ hydroxyl of the ribose and the carboxyl group of Asp237 (Gly230 is the equivalent residue in *ef*CK). In addition, an interaction that requires the presence of a γ-phosphate is missing in the *ef*CK-ADP structure - a salt bridge to Lys216 amino group (Lys209 in *ef*CK). The Lys209 in *ef*CK adopts an alternative conformation to interact with Asp210 carboxylate, a residue that also interacts with Lys271. This second interaction occurs also in *gl*CK between Asp217 and Lys278. Thus, the dispositions of Asp217 (Asp210 in *ef*CK) and the two lysine residues with respect to the nucleotide are pivotal for nucleotide binding and catalysis, consistent with the previous *ef*CK site-directed mutagenesis study [7]. Most dramatically, the D210N mutation reduced *k*_cat_ by over 1000-fold and increased the *K*_m_ value of ADP by 5-fold.

The γ-phosphate in the *gl*CK-AMPPNP structure is located in an equivalent position to one of the four water molecules that coordinates to Mg^2+^ in the *ef*CK-ADP structure, suggesting that the γ-phosphate would also coordinate to Mg^2+^. However, no electron density is associated with Mg^2+^ in the *gl*CK-AMPPNP structure reported here. We attributed the lack of bound divalent ion to the relatively high citric acid concentration in the crystallization solution (0.4 M), a chelating compound that might have reduced the effective concentration of the metal ion. Attempts to obtain crystals with higher magnesium concentration were unsuccessful. We therefore propose that although catalytic activity requires the presence of a divalent cation [20], the metal ion is not required for nucleotide binding in the active site. Our interpretation is consistent with the *ef*CK-ADP structure where Mg^2+^ coordinates solely to the ADP and water molecules. We note that the absence of a divalent ion from protein crystal structures containing AMP-PNP is not unusual. A survey of the Protein Data Bank showed that of >100 protein structures with bound AMPPNP, ∼25% lack an accompanying Mg^2+^.

### Citrate Binding Site

Superposition of the *gl*CK-citrateS and *gl*CK-AMPPNP structures reveals that the citric acid binds adjacent to the AMP-PNP such that the C4–C5 acetyl group of citric acid overlaps with the AMP-PNP’s γ-phosphate group (see Fig. 3C for atom numbering and protein-ligand interactions). In addition, superposition of the *ef*CK-SO_4_ structure with the *gl*CK-citrateS and *gl*CK-AMPPNP structures places the SO_4_^2+^ close to the C6-carboxyl moiety of the citric acid. In both *gl*CK-citrate and *ef*CK-SO_4_ structures the auxiliary domains are in the closed conformations, and the closure partially protects the ligands from bulk solvent. As proposed earlier, the carbamoyl phosphate binds in this site [6], [7]. Note that the citrate binds in the glycerol site identified in the earlier low-resolution crystal structure of *gl*CK, however in the low-resolution structure, all auxiliary domains were disordered [6].

As with the nitrogen atom of the AMP-PNP β,γ-imidophosphate, the C5-carboxylate of citric acid interacts with the backbone nitrogen of Gly13 (Fig. 3A). An analogous interaction occurs in the *ef*CK-SO_4_ structure (Gly11 in *ef*CK), where SO_4_^2+^ binding is also accompanied by a conformational change of residues 11–12 relative to the *ef*CK-ADP structure, resulting in the backbone amides of both residues Gly11 and Asn12 interacting with the sulfate ion. Whereas the sulfate ion binds 4.3 Å away from the ADP’s β-phosphate, the citrate’s C4–C5 acetyl group is placed within a covalent bond distance from the β-phosphate of the AMP-PNP, and there is no conformational difference between the backbone conformation of Gly13 and Asn14 in the *gl*CK-citrate and *gl*CK-AMPPNP structures.

Other CK-citrate interactions that may mimic CK-carbamoyl phosphate interactions include the interaction of the C6 carboxyl group with the backbone amides of Asn55 and Gly56, located at the N-terminus of an α-helix (Fig. 3A&C). A similar “oxyanion hole” is also conserved in the *ef*CK, where it serves as a second anchor for the sulfate anion. The C6 carboxyl group also interacts with Lys131’s amino group, located on the auxiliary domain. Again, a similar interaction between the sulfate and Lys128 is present in the *ef*CK-ADP structure [7]. Finally, the C1 carboxyl group is bridged to Arg163 on the auxiliary domain *via* a water molecule. In the *ef*CK structure, a second bound sulfate ion forms a salt bridge with the equivalent arginine residue (Arg158 in *ef*CK).

### Phosphotransfer Transition State Model

Superposition of the AMP-PNP and citrate bound structures reveals an overlap between the positions of the two ligands. The γ-phosphate of AMP-PNP and the C5 carboxylate group of the citrate are placed such that the β-phosphorous atom of AMP-PNP and one of the carboxylate oxygen atoms of the citrate are separated by 1.7 Å. These two structures provide further support to the previously proposed catalytic mechanism, which involves an in-line nucleophilic substitution at phosphorous [6], [7]. Moreover, the active site lacks an appropriately positioned histidine or aspartic acid residues that may serve as a temporary phosphoryl group acceptor in a two-step phosphotransfer reaction involving a phospho-enzyme intermediate (see reviews of phosphotransfer reaction mechanisms [21], [22], [23]).

**Figure 4:**
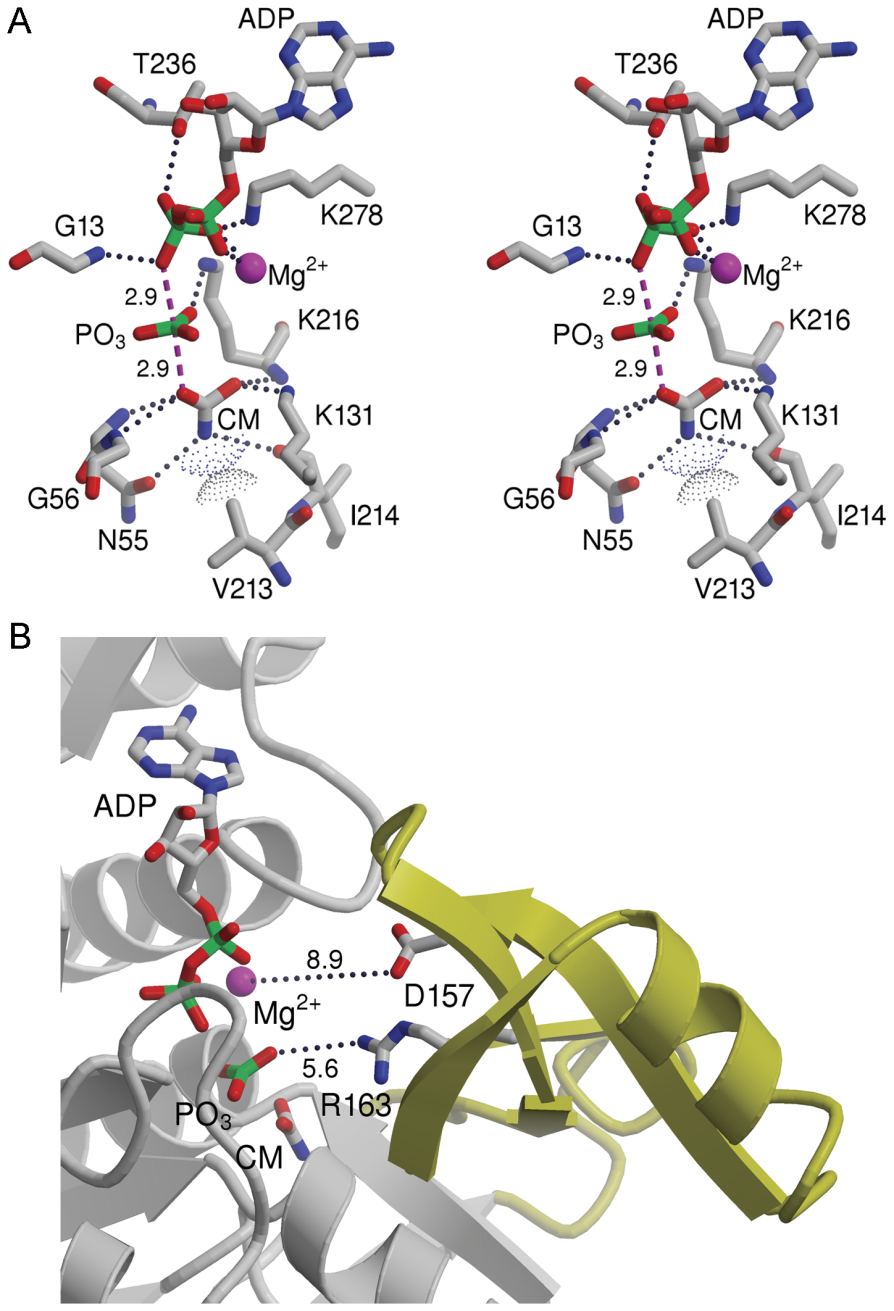
A model of the transition state. (A) Stereoscopic representation showing the key protein and Mg^2+^ interactions with the transition state components. The apical axis between the donor and acceptor oxygen atoms is shown as dashed lines in magenta and the distances from the transferred phosphorous to the donor and acceptor oxygen atoms are 2.9 Å. Atoms are colored as follows: Carbon – gray, Oxygen – red, Nitrogen – blue, Magnesium – magenta. The carbamate is labeled CM (B) The relationship between the transition state and Arg163 and Asp157 on the auxiliary domain (colored in yellow), as defined by the closed conformation of *gl*CK. The side chains of Arg163 and Asp157 project towards the transition state. However, the distances (listed in Å) are too far, allowing bulk solvent access into the active site. Protection of the transition state and prevention of phosphate hydrolysis requires a further closure of the auxiliary domain.

A model of the transition state formed during the phosphoryl group transfer is shown in Fig. 4A. The protein model in this transition state is a composite of two crystal structures; the closed conformation of the auxiliary domain as seen in the *gl*CK-citrate structure and the C-terminal domain’s loop containing residues 245–250 as seen the *gl*CK-AMPPNP structure. The Mg^2+^ was positioned by analogy to the *ef*CK-ADP structure. The location of the AMP-PNP defines the ADP binding site. The ADP-protein interactions correspond to those described above for the AMP-PNP, which include interactions with both the protein backbone and the side chains. The β-phosphate is anchored not only by the interactions with the protein (Gly13 backbone amide, Lys278 amino group, and Thr236 hydroxyl group) but also by coordination to Mg^2+^, as observed in the *ef*CK-ADP structure.

The location of the carbamoyl moiety overlaps with the citrate and is delineated by favorable electrostatic interactions and also by the avoidance of van der Waals clashes. Specifically, the carbamoyl’s nitrogen atom binds adjacent to the side chain of Val213 (Fig. 4A), an invariant residue in all CK sequences (Fig. 2D). Together with the carbamoyl orientation relative to ADP, the restricted position of the carbamoyl’s nitrogen atom defines the location of the carbamoyl phosphate site. Whereas the proximity of the nitrogen atom to Val213 still allows a hydrogen bond interaction of the carbamoyl’s nitrogen atom with the side chain of the invariant Asn55, it prevents the nitrogen atom from forming a strong electrostatic interaction with the backbone carbonyl oxygen of Ile214 (the distance is 3.6 Å). In contrast, the carboxylate group of the carbamoyl moiety forms four favorable interactions. The free oxygen interacts with the amino group of the invariant Lys131, a residue located on the auxiliary domain, and with the backbone amide of Lys216. The phosphoryl group donor oxygen atom is located in an oxyanion hole formed by the backbone amide groups of Asn55 and Gly56 observed in all available CK structures.

The transition state’s planar PO_3_ group is located half-way between the donor and acceptor oxygen atoms, such that the O(donor)-P-O(acceptor) atoms form the apical axis and the PO_3_ oxygen atoms lie in the equatorial plane of a trigonal bi-pyramid centered on the phosphorous. The PO_3_ group interacts with the amino group of the invariant Lys216, as does the AMP-PNP γ-phosphoryl group in the experimental *gl*CK-AMPPNP structure. However, unlike the AMP-PNP’s γ-phosphate, the PO_3_ in the transition state model is located 3.7 Å from Mg^2+^, i.e. too remote for direct coordination to Mg^2+^. The reaction direction towards ATP production may be governed in part by the preference to establish a γ-phosphate-Mg^2+^ coordination.

The proposed transition state model places the phosphoryl donor and acceptor oxygen atoms 5.8 Å apart, with the phosphorous at an equal distance (2.9 Å) to the donor and acceptor. The distance agrees closely with the quoted donor-acceptor oxygen atoms’ distance of 6.0 Å in the model reported by Rubio’s and colleagues [7]. A 2.9 Å long apical P-O distance corresponds to a predominantly dissociative mechanism. Using Pauling’s rule [24], the bond order is 0.02. The dissociative phosphotransfer mechanism has been implicated in previous crystal structures of creatine kinase, glycocyamine kinase, and arginine kinase, where each enzyme formed a complex with a transition state analog [25], [26], [27]. In these structures, the transition state analogs comprised ADP, nitrate (a metaphosphate analog) and the respective substrates. The distances between the nitrate’s nitrogen atom to either donor and acceptor oxygen atoms are ∼3 Å in all these cases, consistent with the distances in the carbamate kinase transition state model.

In contrast to the three phosphagen kinase structures listed above, where the metaphosphate analog, nitrate, is fully buried and protected from bulk solvent, the closed conformations in both *gl*CK and *ef*CK structures do not fully sequester the transition state, allowing bulk solvent to access the metaphosphate. Indeed, only one of the metaphosphate’s oxygen atoms interacts with a protein residue (Fig. 4A). An exposed transition state would be a target for hydrolysis. It is tempting to speculate that the auxiliary domain may close further so that the transition state is fully protected to promote the phosphotransfer reaction and prevent a non-productive hydrolysis. Two invariant residues, Asp 157 and Arg163, are located on the surface of the auxiliary domain exposed to solvent and they could potentially facilitate further closure (Fig. 4B). In a true transition state, Arg163 could interact with the transferred phosphoryl group and Asp157 could perhaps coordinate to the Mg^2+^.

The superposition of the *gl*CK and *ef*CK structures suggests that the transition state would be further protected from bulk solvent if the *gl*CK residues Gly13-Asn14 adjust to generate an oxyanion hole analogous to the oxyanion hole of Gly12-Asn13 in the *ef*CK-SO_4_ structure (invariant residues in all CK sequences). This generates electrostatic interactions between the two protein backbone amides and the remaining free metaphosphate oxygen atom, and also forms hydrogen bonds between Asn14 side chain and both, the metaphosphate and the β-phosphate of ADP. Together, the further closure of the auxiliary domain and the transition of Gly13-Asn14 to form an oxyanion would fully sequester the transferred phosphoryl. Whether such conformational transitions could also induce contraction of the transition state towards a more associative phosphotransfer reaction mechanism remains to be determined.

### Conclusion

The crystal structures of *gl*CK bound with citric acid and with AMP-PNP reveal a dynamic molecule that exhibits substantial conformational flexibility within the same crystal form. The lack of bound Mg^2+^ in the *gl*CK-AMPPNP structure indicates that the divalent cation is not necessary for nucleotide binding, although it is required for catalysis. The transition state model, proposed based on the two crystal structures, is consistent with an in-line phosphotransfer reaction mechanism that is predominantly dissociative. Protection of the transition state from bulk solvent suggests a further closure of the auxiliary domain. Structures of additional complex with a transition state analog, kinetic isotope effect, analysis of stereochemical outcome, and site directed mutagenesis studies of residues postulated to facilitate catalysis and active site closure would help elucidate the catalytic mechanism. With respect to novel antigiardiasis therapeutic approaches, the new structures provide a solid structural basis for drug development and show that new inhibitors may be designed with multiple conformational states of the enzyme in mind.
